# Respiratory Syncytial Virus Interferon Antagonist NS1 Protein Suppresses and Skews the Human T Lymphocyte Response

**DOI:** 10.1371/journal.ppat.1001336

**Published:** 2011-04-21

**Authors:** Shirin Munir, Philippa Hillyer, Cyril Le Nouën, Ursula J. Buchholz, Ronald L. Rabin, Peter L. Collins, Alexander Bukreyev

**Affiliations:** 1 Laboratory of Infectious Diseases, National Institute of Allergy and Infectious Diseases, National Institutes of Health, Bethesda, Maryland, United States of America; 2 Center for Biologics Evaluation and Research, US Food and Drug Administration, Bethesda, Maryland, United States of America; North Carolina State University, United States of America

## Abstract

We recently demonstrated that the respiratory syncytial virus (RSV) NS1 protein, an antagonist of host type I interferon (IFN-I) production and signaling, has a suppressive effect on the maturation of human dendritic cells (DC) that was only partly dependent on released IFN-I. Here we investigated whether NS1 affects the ability of DC to activate CD8+ and CD4+ T cells. Human DC were infected with RSV deletion mutants lacking the NS1 and/or NS2 genes and assayed for the ability to activate autologous T cells *in vitro*, which were analyzed by multi-color flow cytometry. Deletion of the NS1, but not NS2, protein resulted in three major effects: (i) an increased activation and proliferation of CD8+ T cells that express CD103, a tissue homing integrin that directs CD8+ T cells to mucosal epithelial cells of the respiratory tract and triggers cytolytic activity; (ii) an increased activation and proliferation of Th17 cells, which have recently been shown to have anti-viral effects and also indirectly attract neutrophils; and (iii) decreased activation of IL-4-producing CD4+ T cells - which are associated with enhanced RSV disease - and reduced proliferation of total CD4+ T cells. Except for total CD4+ T cell proliferation, none of the T cell effects appeared to be due to increased IFN-I signaling. In the infected DC, deletion of the NS1 and NS2 genes strongly up-regulated the expression of cytokines and other molecules involved in DC maturation. This was partly IFN-I-independent, and thus might account for the T cell effects. Taken together, these data demonstrate that the NS1 protein suppresses proliferation and activation of two of the protective cell populations (CD103+ CD8+ T cells and Th17 cells), and promotes proliferation and activation of Th2 cells that can enhance RSV disease.

## Author Summary

Respiratory syncytial virus (RSV) is a leading cause of pediatric lower respiratory tract disease. RSV has two IFN-I antagonist proteins, NS1 and NS2. In this study, we infected primary human dendritic cells with recombinant RSV from which the NS1 and/or the NS2 genes were deleted, and evaluated effects on the proliferation of autologous T lymphocytes during co-culture *in vitro*. We found that NS1, but not NS2, has a suppressive effect on two cell populations, namely CD103+ CD8+ T cells and Th17 cells, which are known to protect against viral respiratory infections, and a stimulatory effect on Th2 cells, which are involved in enhanced disease caused by RSV. We also provide evidence that these effects are not due to suppressed IFN-I production or signaling in dendritic cells or T cells, and that they likely result from reduced maturation of dendritic cells caused by the NS1 protein.

## Introduction

Following the identification of the NS1 protein of influenza A virus as a type I interferon (IFN-I)-antagonist more than a decade ago [1], such proteins have been identified for many viruses. Their effects on the innate immune system include interference with IFN-I induction, IFN-I-activated signaling, and components of the IFN-I-induced antiviral state (reviewed in [2]). The effects of viral IFN-I antagonists on the adaptive immune response are, however, largely unexplored. Meanwhile, IFN-I is known to have profound effects on key individual components of the adaptive immune system. These effects are diverse and may include both stimulatory and inhibitory roles. For example, several studies have shown that IFN-I promotes maturation of dendritic cells (DC) (reviewed in [3]), although one study demonstrated an inhibitory effect on mouse Langerhans cells, which are DC located in the epidermis [4]. The effect of IFN-I on T cells is also quite complex and depends on various factors including the type of T cells (such as CD8+, CD4+ Th1, or CD4+ Th2), their stage of development, the concentration of IFN-I [5], the experimental system used, and innate inflammatory signals produced during infection, which can be different across various pathogens [6]. For example, while some studies demonstrated that IFN-I stimulates activation and proliferation of T cells [7], [8], [9], [10], others demonstrated an inhibitory effect [11], [12], [13], [14]. In the case of RSV, IFN-I has been demonstrated to inhibit proliferation of CD4+ T cells *in vitro* [15]. Thus, the net effect of viral IFN-I antagonist proteins on the adaptive immune response is difficult to predict since it may be the outcome of opposing (i.e. stimulatory and inhibitory) effects of IFN-I on various individual components of the adaptive immune system.

RSV continues to be the most important viral agent of severe respiratory tract illness in infants and children worldwide and is an important cause of morbidity and mortality in the elderly and severely immunocompromised individuals (reviewed in [16], [17]). An unusual feature of RSV is its capability to re-infect symptomatically (albeit with reduced disease) throughout life without significant antigenic change [18], [19], suggesting an ability of the virus to partially suppress or evade the host's adaptive immune response. Several mechanisms contributing to immune evasion by RSV have been identified. One of them is the expression of the secreted form of RSV G protein, one of the two major neutralization antigens of RSV, which prevents efficient antibody mediated neutralization of the virus [20]. Furthermore, the RSV NS1 and NS2 accessory proteins are known to inhibit the production of IFN-I and type III interferon, and to inhibit signaling resulting from the engagement of the IFN-I receptor (IFNAR) [21], [22], [23], [24], [25], [26]. The two proteins also were demonstrated to inhibit apoptosis [27]. Previously, we analyzed the effect of the NS1 and NS2 proteins on stimulation of human DC by RSV. We reported that the NS1 protein suppresses DC maturation, which was only partly due to suppression of the IFN-I response; furthermore, this effect was somewhat enhanced by the added deletion of the NS2 protein, whereas deletion of NS2 alone had no effect [28].

In the present study, we extend these findings by evaluating the role of the NS1 and NS2 proteins in RSV-specific human T cell responses by co-culturing primary human CD4+ or CD8+ T cells with autologous immature monocyte-derived DC that had been infected with recombinant RSVs lacking the NS1 and/or NS2 proteins. DC derived from primary human monocytes represent an appropriate model for lung-infiltrating DC, since monocytes give rise to mucosal DC [29], which are highly relevant to the immune response to RSV that occurs in pulmonary mucosa [30]. Moreover, inflammation is a typical feature of acute infections with RSV and other respiratory viruses, and monocyte-derived DC are phenotypically and functionally similar to DC located at sites of inflammation *in vivo* [31]. Another important advantage of the human DC-T cell co-cultivation system is that it avoids limitations of the commonly used mouse model for RSV, which is based on a non-natural host that is only semi-permissive for RSV infection. Mice also have some incongruities in their immune system compared to humans; for example, IFN-I activates human and mouse T cells by different mechanisms [32], [33]. In the present study, we identified three major effects of the NS1 protein on T cells that likely play a role in reducing the efficiency of the adaptive immune response to RSV and may contribute to disease severity. We also investigated the role of IFN-I in regulating these effects.

## Results

### Co-culture of virus-infected human DC with autologous CD4+ and CD8+ T cells

**Figure 1:**
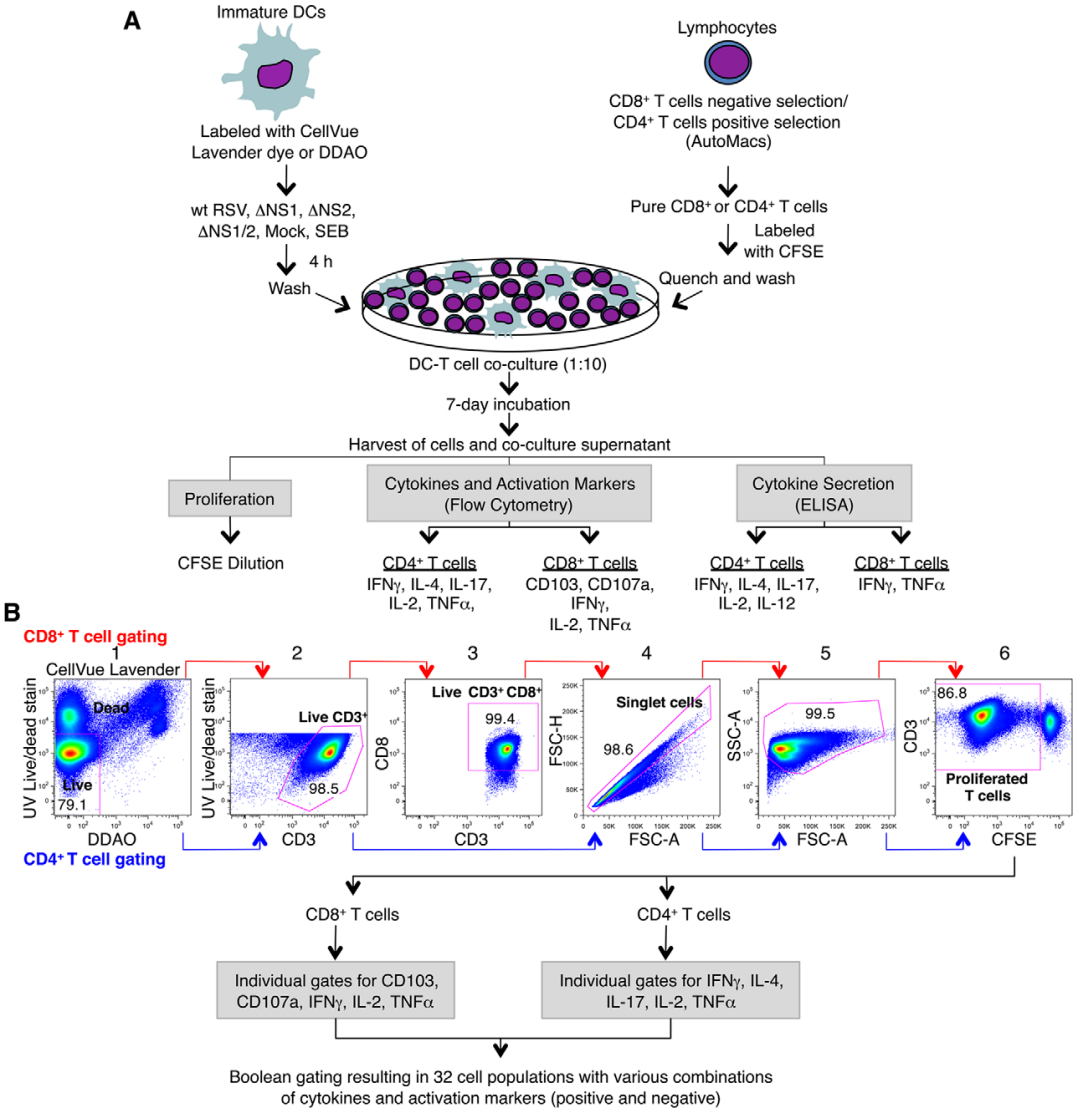
Co-cultivation of human DC and T cells and multi-color flow cytometetry analysis of T cells. **A.** Co-cultivation of human DC pre-infected with wt RSV and its NS1/2 deletion mutants with autologous CD8+ or CD4+ T cells. Immature DC were generated *in vitro* from peripheral blood monocytes and were labeled with CellVue Lavender dye or DDAO. CD8+ and CD4+ T cells were purified from elutriated lymphocytes from the same donors by negative and positive selection, respectively. DC were inoculated with wt RSV or the NS1/2 deletion mutants (MOI of 2 PFU/cell), SEB-treated, or mock-treated for 4 h, and co-cultured with purified CFSE-labeled CD8+ or CD4+ T cells for 7 days. Co-culture supernatants were analyzed for the indicated cytokines by ELISA, and the T cells were stained for the indicated cytokines and activation markers followed by multicolor flow cytometry. **B.** Gating and analysis of CD8+ and CD4+ T cells by flow cytometry. The selected cell subset of each stage is outlined in red: gate 1, live/dead staining versus DC labels (CellVue Lavender or DDAO) to eliminate DC and dead T cells; gate 2, live/dead staining versus CD3 to obtain live CD3+ T cells; gate 3, CD8 versus CD3 (made for only CD8+ T cells) to ensure the CD3+CD8+ phenotype of the CD8+ T cells; gate 4, forward scatter height versus forward scatter area to obtain an unclustered single cell population; gate 5, side scatter area versus forward scatter area to examine cell size and granularity; and gate 6, CD3 versus CFSE to obtain proliferated CD3+ T cells, as detected by dilution of CFSE due to cell divisions. Proliferated T cells were then analyzed for the expression of individual activation markers and intracellular cytokines and also for sub-populations expressing various combinations of them by Boolean gating using FlowJo software.

We investigated the effects of the RSV NS1 and NS2 proteins on the ability of human monocyte-derived DC to activate autologous T cells during co-culture *in vitro* (Fig. 1). DC were infected with 2 plaque forming units (PFU) per cell of wt RSV or RSV that lacks the NS1, NS2, or both genes and expresses enhanced green fluorescent protein (GFP) (ΔNS1, ΔNS2, ΔNS1/2 [28]). The DC were washed and co-cultured with purified autologous CD8+ or CD4+ T cells and incubated for 7 days (Fig. 1A). The T cells were then immunostained for surface markers and intracellular cytokines and analyzed by multi-color flow cytometry (Fig. 1B). Flow cytometry data were analyzed for (i) proliferated CD8+ or CD4+ T cells measured by dilution of carboxyfluorescein diacetate succinimidyl ester (CFSE) intensity due to cell division, (ii) proliferated CD8+ or CD4+ T cells positive for each individual marker, and (iii) proliferated T cell populations positive for one or more markers and negative for the other markers, resulting in a total of 32 combinations (Fig. 1B). Since essentially all adults have been exposed to RSV due to prior natural infections, the proliferating T cells would primarily represent activation of memory cells. The possible presence of trace numbers of RSV-specific naïve T cells would not affect the study. We note that viruses lacking the NS1 and/or NS2 proteins are similar to wt virus with regard to their efficiency of infection of human DCs and have only a modest reduction in viral gene expression [28]. Thus, changes in immune cells associated with the deletions (below) can be attributed to the absence of the NS1/2 genes rather than substantial changes in viral growth or gene expression.

### The NS1 protein suppresses the activation of CD8+ T cells positive for the mucosal tissue homing molecule CD103 (α_E_β_7_ integrin)

**Figure 2:**
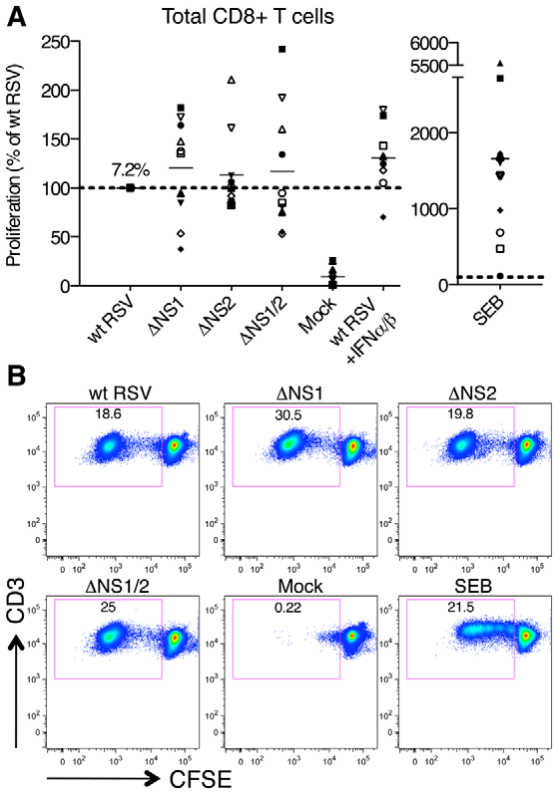
Proliferation of CD8+ T cells during co-cultivation with autologous DC that had been pre-infected with wt RSV or its NS1/2 deletion mutants. **A.** Extent of CD8+ T cell proliferation in response to co-culture with DC pre-infected with wt RSV or the NS1/2 deletion mutants, normalized to results with wt RSV assigned the value of 100% (dotted line). A total of 10 donors were analyzed, indicated individually by symbols, with all viruses compared in parallel for each donor. The horizontal bars represent the mean values. For wt RSV, the mean extent of CD8+ T cell proliferation (7.2%) is expressed as a percentage of the total CD8+ T cell population. No statistically significant difference between T cells co-cultivated with DC infected with various viruses was found. **B.** Example of primary flow cytometry data using cells from one representative donor; the percentage of each proliferated population is indicated.

Co-cultivation of wt RSV-infected DC with CD8+ T cells resulted in significant proliferation of T cells, which was negligible with mock-infected DC (Fig. 2). As compared to wt RSV, deletion of the NS1 gene resulted in an increased proliferation of CD8+ T cells using cells from 6 donors and reduced proliferation with cells from 4 donors. Deletion of the NS2 gene resulted in an increased proliferation of cells from only 2 of 10 donors (Fig. 2). On average, deletion of the NS1, NS2, and NS1 and NS2 together resulted in an increase in mean CD8+ T cell proliferation of 20, 13 and 17%, respectively, as compared to wt RSV, and none of these differences were significant. Thus, the effect of NS1 and/or NS2 deletion on total CD8+ T cell activation was modest and inconsistent.

**Figure 3:**
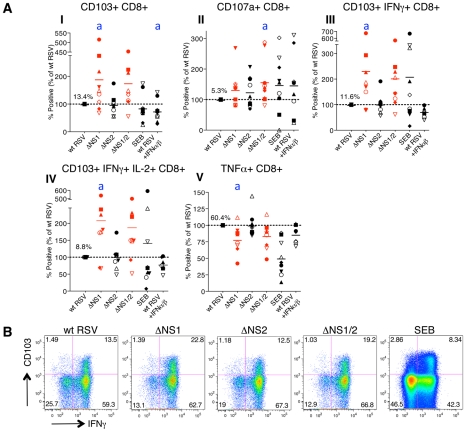
The NS1 protein suppresses activation and proliferation of CD103+ CD8+ T cells. **A.** Percentages of proliferating CD8+ T cells positive for the indicated markers of activation, measured after co-culture with autologous DC that had been pre-infected with the ΔNS1 (shown in red), ΔNS2 or ΔNS1/2 RSV (red) deletion mutants, as compared to cells from the same donors co-cultured with DC pre-infected with wt RSV (which was assigned a value of 100%, dotted line). In addition, a duplicate of the wt RSV co-culture was prepared containing 133 IU each of exogenously added IFNα2a and IFNβ, similar to the amounts that are produced by DC infected with the ΔNS1 virus [28]. The markers of activation that were analyzed are indicated on the top of each panel. A total of 9 donors were analyzed, indicated individually by symbols, and the horizontal bars represent the mean values. In each panel, the mean amount of each subpopulation as a percentage of the total proliferating CD8+ T cells is shown for wt RSV. Since only proliferating cells were analyzed, cells co-cultivated with mock-infected DC were not analyzed due to their near-absence of proliferation (see Fig. 2A, B). For the ΔNS1 and ΔNS1/2 viruses, statistical significance compared to wt RSV is indicated by letter a, P<0.05. Statistical significance is also indicated for wt RSV treated with IFNα2a and IFNβ compared to un-treated wt RSV: a, P<0.05 (indicated for panel I only). **B.** Examples of the primary flow cytometry data with cells from one representative donor; the percentage of each proliferated population is indicated.

We then evaluated the effects of the NS1 and NS2 deletions on activation of specific subsets of CD8+ T cells by multicolor flow cytometry. The analysis involved staining for two T cell surface proteins: CD103 (the α_E_ subunit of the α_E_β_7_ integrin), which is preferentially expressed by CD8+ T lymphocytes present in mucosal tissue, directs lymphocytes to mucosal tissues by binding to the epithelial cell marker E-cadherin [34], [35], [36], and triggers cytolytic activity of CD8+ T cells in lung tissue [37], and CD107a, which is a marker of de-granulation of CD8+ CTL [38]. We also analyzed intracellular staining of the cytokines IFNγ, IL-2, and TNFα, which are considered markers of CD8+ CTL activation [39]. We found that deletion of the NS1 gene alone or in combination with the NS2 gene resulted in a dramatic increase in the number of proliferating CD8+ cells that express CD103 (Fig. 3A, panel I). In addition to this increase in the number of CD103+ cells, deletion of NS1 and NS1/2 was also accompanied by an increase in the median fluorescence intensity (MFI) of CD103 expression of up to 32% as compared to wt RSV, an increase that was statistically significant in most, but not all cases (Fig. S1A). In addition, deletion of NS1 or NS1/2 resulted in a mean increase of 30% and 58%, respectively, in total CD8+ T cells positive for CD107a, although in case of the NS1 deletion, the increase was not statistically significant (Fig. 3A, panel II). The presence of this degranulation marker presumably is a consequence of cytotoxic activity of CD8+ T cells against the DC in the co-culture, and thus is a marker for activation. Incidentally, cytotoxic attack on DC is not necessarily an aberrant activity, and indeed has been suggested to occur *in vivo* as a means of down-regulating further T cell activation [40]. It also should be noted that CD107a transiently appears on cell surface and is rapidly internalized following degranulation [41], and hence the level of CD107a observed on day 7 probably is a substantial underestimate of the total amount of degranulation occurring during the 7 day incubation. Deletion of NS1 or NS1/2 also resulted in substantial increases in CD8+ cells that were positive for CD103 plus IFNγ (Fig. 3A, panel III and 3B) and CD103 plus both IFNγ and IL-2 (Fig. 3A, panel IV). In contrast, deletion of the NS2 gene alone did not alter the expression of these various markers.

Incidentally, deletion of NS1, but not NS2, also was associated with a down-regulation of the number of total CD8+ T cells secreting TNFα in most of the donors (Fig. 3A, panel V). As a possible explanation of this effect, deletion of NS1 is associated with increased secretion of IFN-I by RSV-infected DC [28], and IFN-I has been shown to suppress the production of TNFα [42]. Data are conflicting as to whether TNFα plays a role in CD8+ CTL-mediated lysis of target cells [43], [44]. Perhaps more importantly, TNFα down-regulates memory CD8+ T cell responses by limiting the duration of the CTL effector phase and magnitude of CD8 T cell memory response [45].

Taken together these data suggest that the RSV NS1 protein reduces the number of CD8+ T cells positive for the mucosal tissue homing molecule CD103, reduces activation of these cells, as measured by expression of the de-granulation marker CD107a and IFNγ, and increases the number of CD8+ T cells positive for TNFα.

### The NS1 protein skews the phenotype of CD4+ T cells towards Th2 and reduces activation and proliferation of Th17 and Th17/Th1 cells

**Figure 4:**
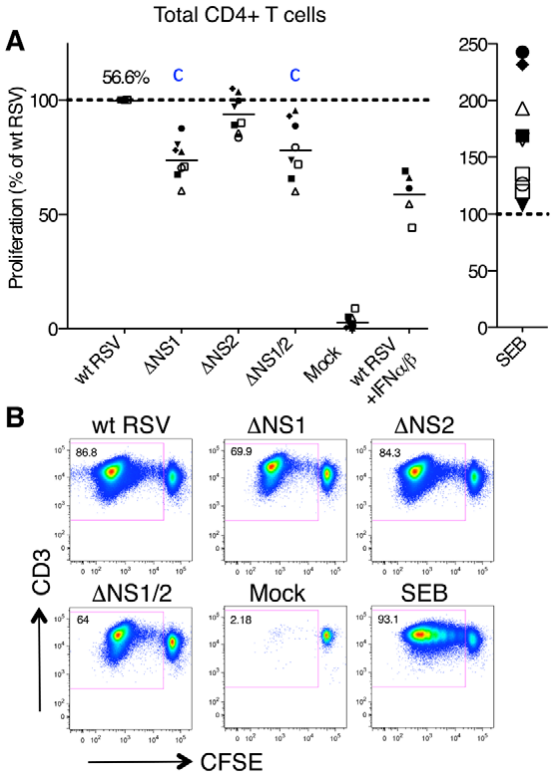
Proliferation of CD4+ T cells during co-cultivation with autologous DC pre-infected with wt RSV or its NS1/2 deletion mutants. **A.** Extent of CD4+ T cell proliferation in response to DC infected with wt RSV or the NS1/2 deletion mutants, normalized to results with wt RSV assigned the value of 100% (dotted line). In addition, duplicate of wt RSV co-cultures were prepared containing 133 IU/ml each of exogenously added IFNα2a and IFNβ. A total of 8 donors were analyzed, represented individually by symbols, and the horizontal bars represent mean values. For wt RSV, the extent of CD4+ cell proliferation as a percentage of the total CD4+ population is indicated. For the ΔNS1 and ΔNS1/2 viruses, statistical significance compared to wt RSV is indicated by the letter c, P<0.001. **B.** Example of primary flow cytometry data for cells from one representative donor; the percentage of each proliferated population is indicated.

We also evaluated the effects of deleting the NS1 and NS2 proteins on the activation of CD4+ T cells using the co-culture system. First, we compared the levels of proliferation of total CD4+ T cells following co-culture with autologous DC pre-infected with wt RSV or the NS1 and/or NS2 deletion mutants using the same experimental design as that used for CD8+ T cells (Fig. 1). Abundant proliferation of RSV-specific CD4+ T cells was detected by CFSE dilution assay when the cells were co-cultivated with DC pre-infected with wt RSV, whereas there was a near-absence of proliferation in response to mock-infected DC (Fig. 4). Deletion of the NS1 gene resulted in a significant reduction in the proliferation of CD4+ T cells (Fig. 4A, B): the effect was observed in all samples and mean reduction associated with deletion of the NS1 and NS1/2 genes was 26% and 22%, respectively. In contrast, deletion of the NS2 gene alone was associated with no change or only a marginal reduction of proliferation (Fig. 4A). Thus, the NS1 protein, but not NS2, promotes CD4+ T cell proliferation.

**Figure 5:**
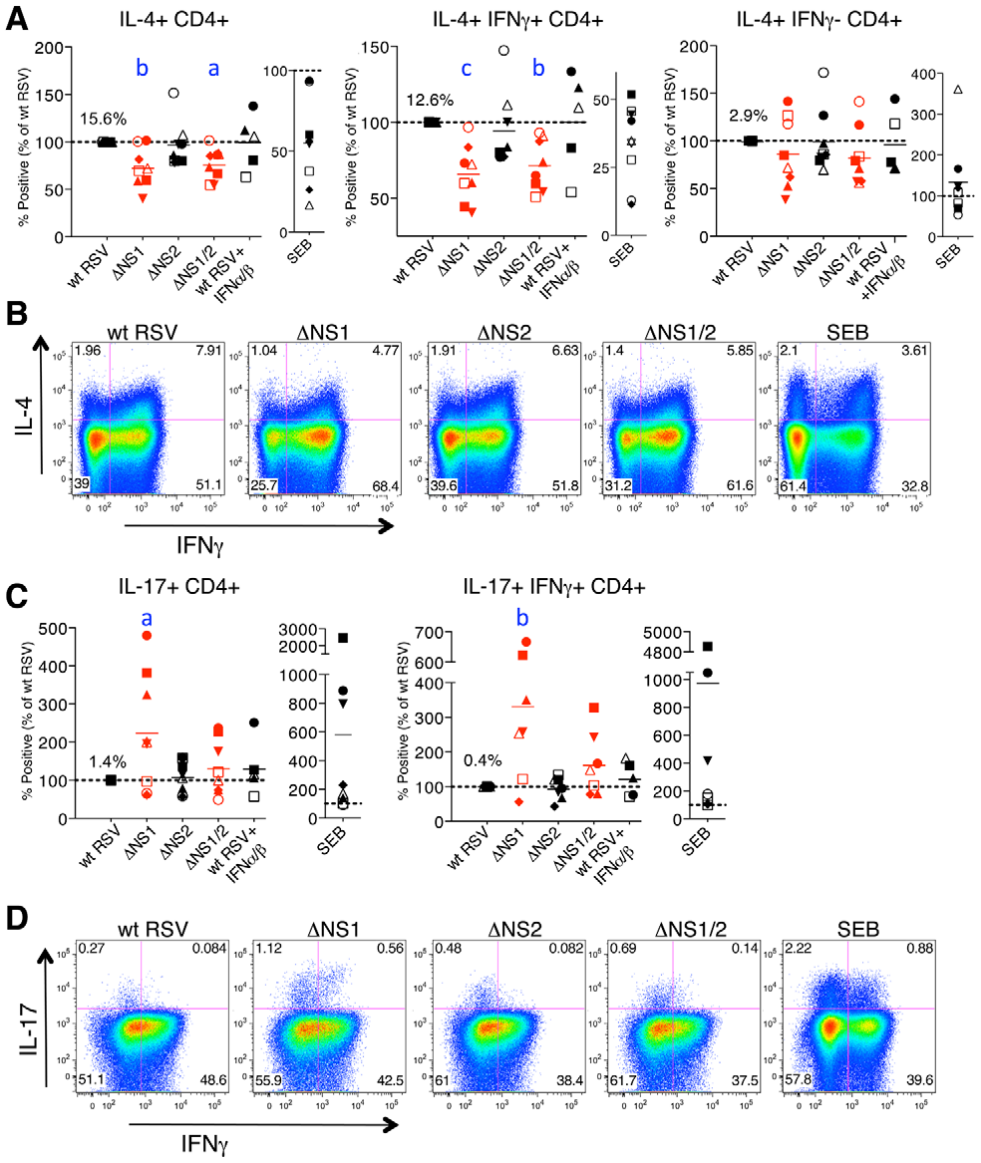
The NS1 protein affects the polarization of CD4+ T cells. **A, B.** The NS1 protein shifts the phenotype of CD4+ T cells towards Th2. **A.** Percentages of proliferating CD4+ T cells positive for the indicated markers of activation after co-culture with autologous DC that had been pre-infected with the ΔNS1 (red), ΔNS2, and ΔNS1/2 (red) deletion mutants, as compared to the cells from the same donors co-cultivated with DC pre-infected with wt RSV (assigned a value of 100%, doted line). **B.** Examples of primary flow cytometry data using cells from one representative donor, with the percentage of each population indicated. **C, D.** The NS1 protein reduces activation and proliferation of Th17 cells. **C.** Percentages of proliferating CD4+ T cells positive for the indicated markers of activation after co-culture with autologous DC that had been pre-infected with the indicated virus, as compared to wt RSV in cells from the same donor as 100%. **D.** Examples of primary flow cytometry data using cells from one representative donor, with the percentage of each population indicated. In A-D, cells co-cultivated with mock-infected DC were not analyzed due to the near-absence of proliferation (see Fig. 4). In addition, duplicate of wt RSV co-cultures were prepared containing 133 IU/ml each of exogenously added IFNα2a and IFNβ. In A and C, the markers of activation that were analyzed are indicated on the top of each panel. A total of 8 donors were analyzed, represented by individual symbols, and the horizontal bars indicate mean values. In each panel, the amount of each subpopulation as a percentage of the total proliferating CD4+ T cells is shown for wt RSV. For the ΔNS1 and ΔNS1/2 viruses, the statistical significance of the difference to wt RSV is indicated in blue as follows: a, P<0.05; b, P<0.01, c, P<0.001.

We also investigated the effects of the NS1 and NS2 proteins on the cytokine profiles of proliferating CD4+ T cells from the co-culture experiments described above by intracellular cytokine staining of T cells and multi-color flow cytometry. The analyzed cytokines included markers for the Th1 (IFNγ), Th2 (IL-4), and Th17 (IL-17) subsets, as well as general markers of activation (IL-2 and TNFα). Unexpectedly, we found a sizable population of CD4+ T cells positive for both IFNγ and IL-4, which made up 12.6% of total CD4+ T cells proliferating in response to stimulation with wt RSV-infected DC and also accounted for most of the IL-4+ population (Fig. 5A, B). CD4+ T cells positive for both IFNγ and IL-4 were previously identified in mice and have been cloned and characterized *in vitro* [46]. A recent study demonstrated that infection of mice with lymphocytic choriomeningitis virus reprogrammed otherwise stably committed Th2 cells to adopt an IL-4+IFNγ+ “Th2+1” phenotype that was maintained *in vivo* for months [47]. However, to the best of our knowledge a similar human Th cell population has not yet been reported [48]. In the present study, control experiments excluded the possibility that the Th1/Th2 phenotype of CD4+ T cells was the result of fluorochrome spectral overlap: specifically, the IL-4+ IFNγ+ double positive cells were observed only with antibodies to both IFNγ and IL-4 and were not observed when the cells were stained with all antibodies while omitting those for IFNγ or IL-4 (Materials and Methods and Fig. S2B).

Deletion of the NS1 gene alone or with the NS2 gene, but not deletion of NS2 alone, resulted in a significant reduction in the number of cells secreting IL-4, including total IL-4+ cells and those positive for both IL-4 and IFNγ (Fig. 5A, B) in most of the donors. Furthermore, analysis of IL-4 expression showed that deletion of NS1, but not NS2, also resulted in reduced IL-4 MFI for total IL-4+ as well as IL-4+IFNγ+ cells (Fig. S1B). No change was observed in the total number of IFNγ or IL-2 secreting cells (not shown). These results suggest that the NS1 protein acts as a factor promoting the Th2 response, acting to both shift the bias toward Th2 and to promote proliferation. The reduced percentages of not only IL-4+ but also IL-4+IFNγ+ cells are consistent with our previous studies demonstrating that the induction of a Th2 response during RSV infection in mice results in an increased production of not only the Th2 cytokines, but also of the Th1 cytokine IFNγ [49], as well as the observation by others that the increased production of Th2 cytokines in atopic children is not associated with a reduced level of IFNγ [50].

Deletion of the NS1 gene or both the NS1 and NS2 genes, but not deletion of NS2 alone, also resulted in elevated levels of total IL-17+ cells, as compared to the levels associated with wt RSV, by 125% and 33%, respectively (Fig. 5C, D). Interestingly, we detected a substantial population of the recently described human Th17/Th1 cells positive for both IL-17 and IFNγ [51], and confirmed their double positive phenotype by staining with all antibodies while omitting those for IFNγ or IL-17 (Fig. S2C). The percentage of this cell population was also increased due to deletion of NS1 or both the NS1 and NS2 genes, and the increases were even more dramatic than for total IL-17+ cells, 233% and 64%, respectively. These data indicate that the NS1 protein also antagonizes or alters the adaptive immune response by suppressing the number of Th17 cells, a subset that has recently been shown to be involved in clearance of viral infections *in vivo* (Discussion). Unlike CD8+ T cells, CD4+ T cells demonstrated no change in the percentage of TNFα+ cells on deletion of NS1 or NS2 genes (not shown).

### Role of IFN-I in the NS1 mediated changes in the activation and proliferation of CD8+ and CD4+ T cells

As noted (Introduction), we previously showed that the NS1 protein suppresses DC maturation, due in part to antagonism of IFN-I production and signaling [28]. IFN-I also affects T cell activation, polarization, and proliferation (Introduction, Discussion). Therefore, the suppression of CD103+CD8+ T cells and Th17 cells and enhancement of Th2 cells by NS1 might be mediated by either or both of the two following mechanisms. First, they might result from reduced production of IFN-I by DC, resulting in reduced IFN-I signaling in T cells. Second, they might result from a reduced level of DC maturation, thereby reducing the ability of the DC to activate T cells.

**Figure 6:**
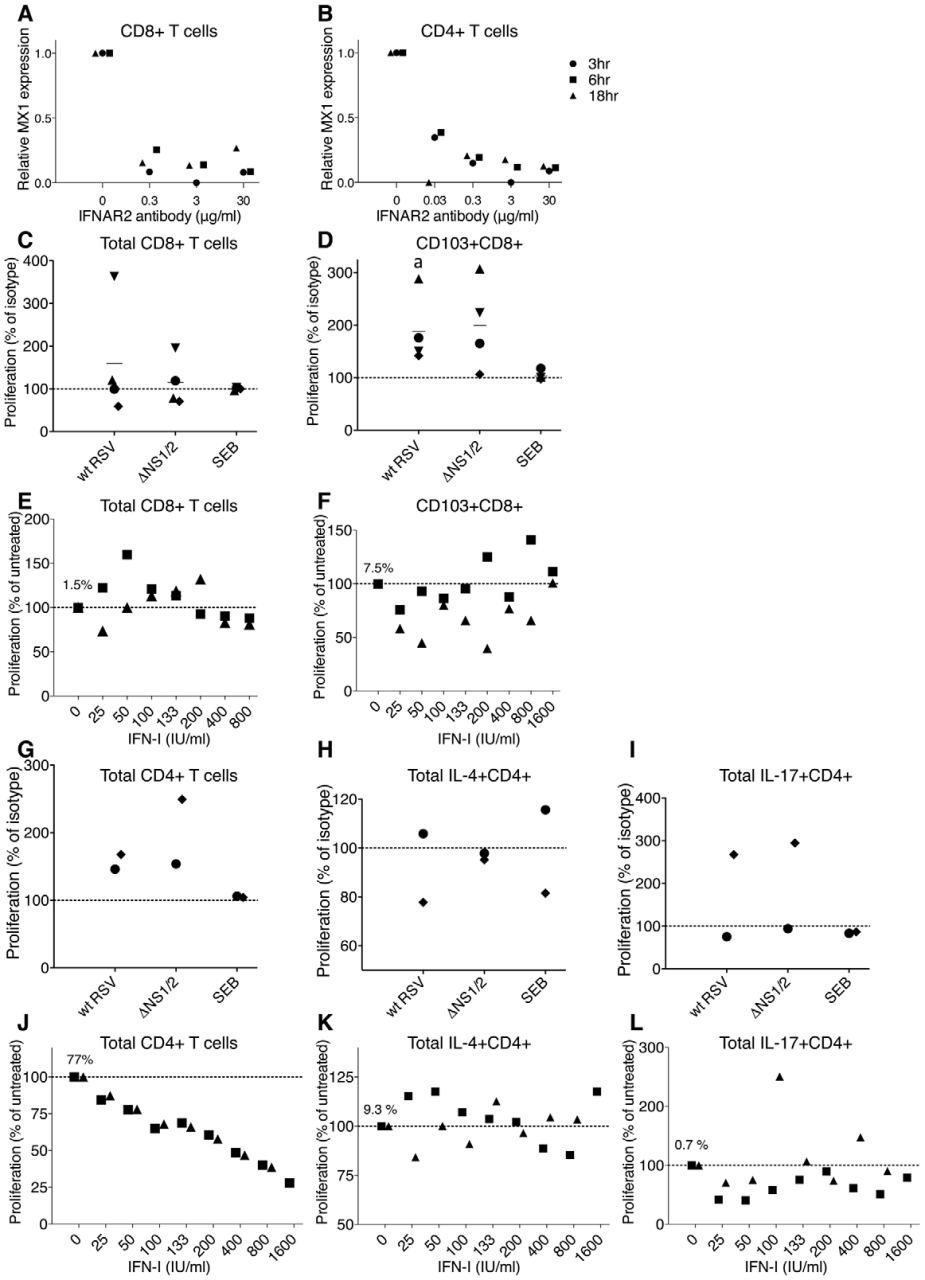
Role of IFN-I in the proliferation of CD8+ and CD4+ T cells. **A, B.** Effectiveness of an IFNAR2-blocking antibody in inhibiting IFN-I signaling. CD8+ (A) and CD4+ (B) T cells from 3 donors were incubated with the indicated concentrations of the IFNAR2 blocking antibody for 2 h, followed by stimulation with 100 IU/ml of IFNα2a. Cells were harvested at 3, 6, and 18 h post-IFN-I treatment, MX1 expression was determined by QRT-PCR and reported as relative expression normalized to cells exposed to IFN-I without antibody. **C, D.** Effect of IFNAR2-blocking antibodies on proliferation of total CD8+ cells (C) and CD103+CD8+ cells (D). The IFNAR2-blocking antibodies or isotype control antibodies were added to CD8+ T cells 1 h prior to their mixing with DC pre-infected with wt RSV or ΔNS1/2 RSV, and the antibodies remained during the co-culture. The level of CD8+ T cell proliferation was normalized to that for cells from the same donor incubated with isotype control antibodies, which was assigned the value of 100% (dotted line). Four donors were analyzed. The statistical significance of the difference between co-culture treated with IFNAR2-blocking antibody compared to its isotype control is indicated by the letter a, P<0.05. **E, F.** Effect of exogenously added IFN-I on the proliferation of total CD8+ cells (E) and CD103+CD8+ T cells (F). IFNα2a and IFNβ, each at the indicated concentration (IU/ml), were added at the beginning of co-culture of CD8+ T cells and DC that had been pre-infected with wt RSV. The amount of CD8+ T cell proliferation in each IFN-I-treated culture was normalized relative to untreated cells, which were given the value of 100% (dotted line). Two donors were analyzed. In panels E and F, the amount of proliferating CD8+ (1.5%) or CD103+CD8+ (7.5%) T cells, respectively, as a percentage of the total CD8+ T cells, is shown for wt RSV lacking exogenous IFN-I treatment. **G, H, I.** Effect of IFNAR2 blockade on the proliferation of total CD4+ T cells (G) or the indicated subpopulation (H and I); representative data from 2 donors. CD4+ T cells were treated with IFNAR2-blocking antibodies or isotype control antibodies for 1 h prior to their mixing with DC pre-infected with wt RSV or ΔNS1/2 RSV, with the antibodies present during the co-culture. The levels of proliferated CD4+ T cells in G, H and I are normalized to that for cells from the same donor treated with isotype control antibodies (100%, dotted line). **J, K, L.** Effect of exogenously added IFN-I on proliferation of total CD4+ T cells (J) or the indicated subpopulation (K and L); representative data from 2 donors. IFNα2a and IFNβ, each at the indicated concentration (IU/ml), were added at the beginning of co-culture of CD4+ T cells and DC that had been pre-infected with wt RSV. The amount of CD4+ T cell proliferation in each IFN-I-treated culture was normalized relative to untreated cells, which were given the value of 100% (dotted line). In panels J, K and L, the mean number of proliferating CD4+ cells as percentages of the total CD4+ T cells (77%, 9.3% and 0.7%, respectively) are shown for wt RSV lacking exogenous IFNα2a and IFNβ.

As a first step in evaluating these possibilities, we investigated the role of IFN-I production and signaling in co-culture experiments in which IFN-I signaling was blocked with an antibody specific to the IFNAR beta subunit (IFNAR2). To determine the effectiveness of the IFNAR2-specific antibody in inhibiting IFN-I signaling, CD8+ (Fig. 6A) and CD4+ (Fig. 6B) T cells were incubated for 2 h with a range of antibody concentrations from 0.3 (Fig. 6A) or 0.03 (Fig. 6B) to 30 µg/ml and then challenged with 100 IU/ml of IFNα2a. The T cells were harvested at 3, 6, and 18 h post-IFN-challenge and the expression of the IFN-I-inducible Mx1 (Fig. 6A and B) and ISG-56 (not shown) genes were analyzed by quantitative RT-PCR (QRT-PCR). This showed that the antibody effectively suppressed induction of either gene even at a concentration that was one-hundredth (Fig. 6A) or one-thousandth (Fig. 6B) of that used in subsequent blockade experiments.

We then investigated the role of IFN-I in the effects on CD8+ T cells in co-cultures. In preliminary experiments, we treated the DC or T cells separately with IFNAR2-blocking antibody for various times, washed away free antibody, and performed co-culture. However, we found that we were unable to detect effects of the antibody blockade on T cell activation (not shown). This perhaps was not surprising, since new surface IFNAR2 molecules likely appear during the long co-culture due to receptor recycling [52] and proliferation. Therefore, we modified the protocol so that the IFNAR2-blocking antibody would be present throughout the co-culture incubation. One unavoidable limitation of this experimental design is that it would not allow us to distinguish directly between effects on DC versus T cells. For these experiments, the IFNAR2-blocking antibody, or an isotype control antibody, was added at a 2-fold concentration (60 µg/ml) to CD8+ T cells, and the cells were incubated for 1 h at 37°C, mixed with an equal volume of autologous DC that had been pre-infected for 4 h with wt RSV or ΔNS1/2 RSV or mock infected, and co-cultured with the antibody present. In one of the four donors, the blockade resulted in a substantial increase in the proliferation of CD8+ T cells compared to the isotype control antibody, and in the other donors, there was either no effect or a moderate reduction of proliferation (Fig. 6C). Thus, a consistent effect was not observed, and usually there was little effect. We next analyzed the effect of IFNAR2 blockade on the proliferation of the CD103+CD8+ T cells, the population most significantly up-regulated by deletion of the NS1 protein, as previously shown in Fig. 3. Somewhat surprisingly, this showed that the blockade increased the percentage of CD103+ cells, an effect that occurred with either virus (Fig. 6D). This is the opposite of the effect that would have been expected if the increase in CD103+CD8+ T cells observed in Fig. 3 for the NS1 and NS1/2 deletion viruses had been due to increased IFN-I production and signaling.

Next, we evaluated the effects of exogenously added IFN-I on CD8+ T cell activation and proliferation. Published studies have variously demonstrated stimulatory or anti-proliferative effects of IFN-I on CD8+ T cells (Introduction). We added increasing amounts of an equal mixture of IFNα2a and IFNβ at the beginning of co-cultivation of wt RSV-infected DC with CD8+ T cells. This resulted in inconsistent effects between the two donors, with small, inconsistent increases or decreases in proliferation at various doses. We next analyzed the CD103+CD8+ T cell population and found that exogenous IFN-I reduced, rather than increased, its percentage, although in cells from one donor this effect was observed only at lower concentrations of IFN-I (Fig. 6F). This is consistent with the antibody blockade results described above in Fig. 6D. An inhibitory, rather than stimulatory, effect of exogenous IFN-I on total CD103+ cells also was observed for most of the donors in the experiments shown in Fig. 3A panel I, in which a single dose of 133 IU/ml each of both IFNα2a and IFNβ was added (similar to the concentration produced by DC infected with the ΔNS1 virus [28]), with the inhibition being statistically significant (P<0.05). The finding that IFN-I and IFN-I signaling suppress rather than stimulate the proliferation of CD103+CD8+ T cells indicates that the effects of NS1 deletion involve a mechanism other than increased IFN-I production and signaling.

Next, we investigated the role of IFN-I in the effects on CD4+ T cells in the co-culture system. We added IFNAR2-blocking antibodies to CD4+ T cells 1 h prior to their co-cultivation with DC that had been pre-infected with wt RSV or ΔNS1/2 RSV, as described above for CD8+ T cells. With either virus, the blockade of IFN-I signaling during co-culture resulted in an increased proliferation of CD4+ T cells (Fig. 6G). However, the subpopulations that were positive for IL-4 or IL-17 did not consistently change with the IFNAR2 blockade (Fig. 6 H and I, respectively). We note that, if increased IFN-I production and signaling indeed had been responsible for the Th2 and Th17 effects observed in Fig. 5, we would have expected to observe an increase in IL-4+ T cells in Fig. 6H and a decrease in IL-17+ T cells in Fig. 6I, which were not observed.

We then examined the effect of exogenously added IFN-I on CD4+ T cell activation. Increasing amounts of a mixture of equal quantities of IFNα2a and IFNβ were added at the beginning of co-culture of wt RSV-infected DC and CD4+ T cells, as was done earlier with CD8+ T cells (above). Addition of the IFN-I cocktail resulted in a dose-dependent reduction in the proliferation of CD4+ T cells (Fig. 6J), which is consistent with our previously published study [15]. However, analysis of individual populations of CD4+ T cells demonstrated a lack of consistent effect on IL-4+ cells (Fig. 6K), and a modest decrease in IL-17+ cells that was not dose-dependent (Fig. 6L). We note that, if increased IFN-I production and signaling had been responsible for the Th2 and Th17 effects observed in Fig. 5, we would have expected to observe a decrease in IL-4+ T cells in Fig. 6K and an increase in IL-17+ T cells in Fig. 6L, which were not observed.

In summary, IFN-I production and signaling in the co-cultures had effects on proliferation of the total population of CD4+ T cells. However, the three major effects associated with the presence of the RSV NS1 protein, namely the reduced proliferation and activation of CD103+CD8+ T cells, the reduced activation and proliferation of Th17 cells, and the skew in the Th1/Th2 balance towards Th2, do not appear to arise from direct effects of IFN-I signaling during co-culture.

### RSV NS1/2 proteins suppress multiple factors of DC maturation that are only partly regulated by secreted IFN-I

**Figure 7:**
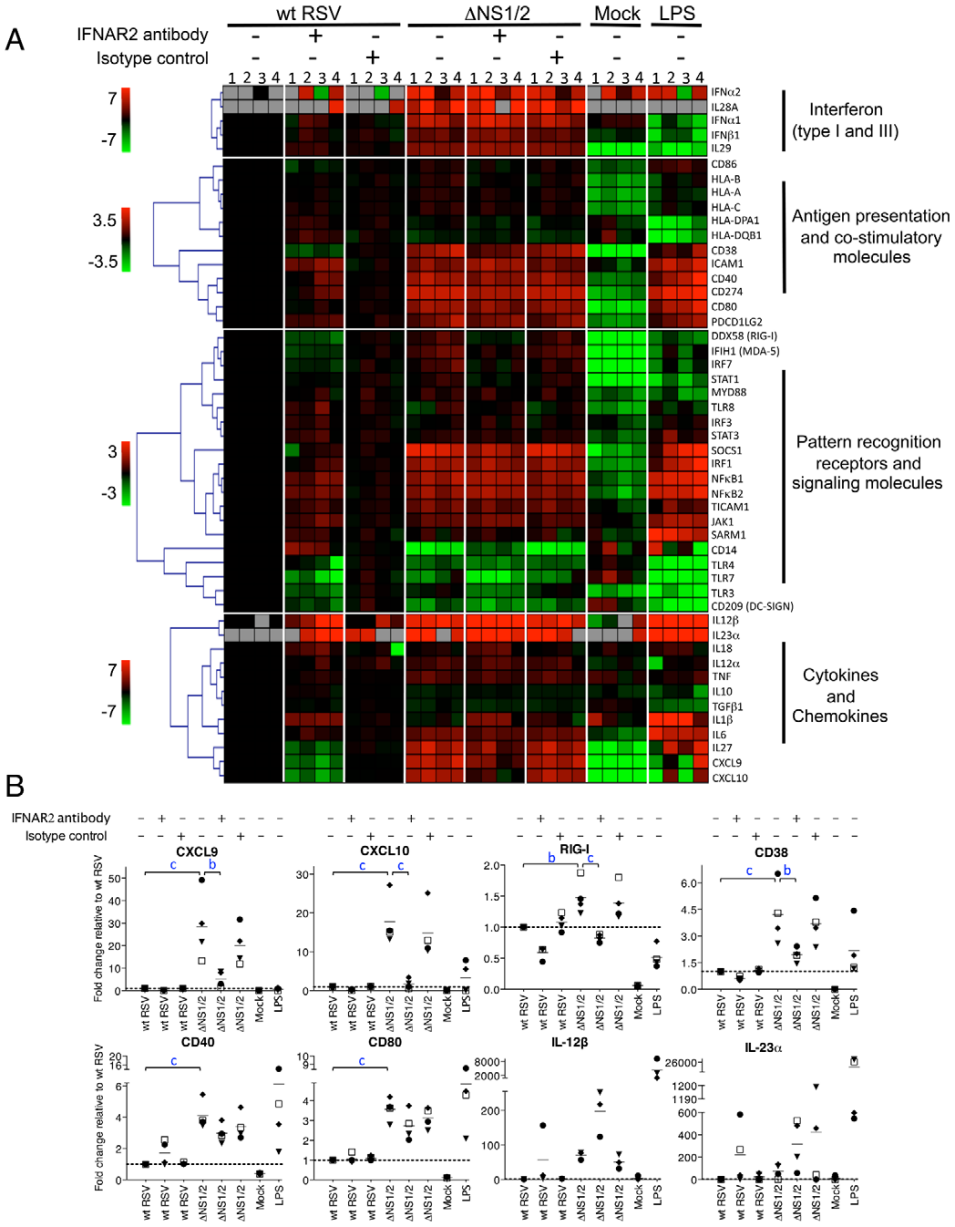
Effect of NS1/2 deletion and IFNAR2 blockade on the transcriptional induction of DC genes. **A.** QRT-PCR analysis of RNA isolated from DC infected with wt RSV or ΔNS1/2 RSV and treated with IFNAR2 antibody, or an isotype control, or treated with LPS or mock. Total cellular RNA was harvested 48 h post-infection and analyzed by QRT-PCR for the indicated mRNAs using a microfluidic gene card. The expression fold change for each treatment was calculated relative to the calibrator, i.e., DC infected with wt RSV without the antibody treatment, converted to log_2_ and used for hierarchical clustering to obtain a heat map. The data from four individual donors are shown (indicated by numerals 1, 2, 3, and 4 on top of the heat map). Each row represents expression status of one gene with the gene name indicated on the right of each row. Genes were divided into four major functional categories indicated on the right of the heat map. Each functional group was clustered separately with the color bar shown on the left of the heat map representing the color range and the corresponding expression fold change indicated by it (red, up-regulated; green, down-regulated; black, no change; grey, mRNA not detected). **B.** Linear plots of the changes in the concentrations of mRNA for CXCL9, CXCL10, RIG-I, CD38, CD40, CD80, IL-12β, and IL-23α, from the experiment in part A. The 4 donors are indicated individually by symbols, and the horizontal bars represent the mean values. The y-axis shows expression fold change relative to wt RSV not treated with antibody. The statistical significance of difference was determined by repeated measures ANOVA with Tukey's multiple comparison post test and the significance of the difference between ΔNS1/2 and wt RSV or between ΔNS1/2 and ΔNS1/2 pretreated with IFNAR2 antibody is indicated in blue as b, P<0.01 and c, P<0.001.

We also investigated the effects of IFN production and signaling on DC. We treated DC from four donors with the IFNAR2 blocking or isotype control antibody for 2 h and then infected them with wt RSV or ΔNS1/2 RSV followed by 48 h incubation in the presence of the blocking antibody. The cells were harvested, total RNA was purified, and mRNAs encoding 49 proteins relevant to DC maturation and function were analyzed by QRT-PCR using a microfluidic gene card format. The results are displayed as a heat map (Fig. 7A) and in some cases in linear plots (Fig. 7B).

We found that deletion of the NS1 and NS2 genes resulted in a dramatic transcriptional up-regulation in a number of mRNAs including ones encoding type I and type III IFNs and other cytokines, molecules involved in antigen presentation and signaling, co-stimulatory factors, and pattern recognition receptors. The IFNAR2-blocking antibody strongly reduced the expression of the IFN-I-stimulated genes CXCL9, CXCL10, and RIG-I (Fig. 6A, B), suggesting that the blockade of IFN-I signaling was effective. The strong IFNAR2 blockade did not reduce the expression of IFNα mRNA (Fig. 6A); also, ELISA analysis of secreted IFNα in the medium demonstrated a 14-fold greater mean concentration for ΔNS1/2 RSV compared to wt RSV (data not shown). This observed expression of IFNα in the face of the IFNAR2 blockade presumably arises by a mechanism that is not dependent on signaling from the IFNAR, such as through activation of IRF7 or IRF5 that are constitutively expressed in human monocyte-derived DC [53]. We found that the IFNAR2 blockade resulted in a partial transcriptional down-regulation of DC maturation markers including CD38, CD40 and CD80 (Fig. 6A, B), in a dose-dependant manner (data not shown). This is consistent with our previous study, which showed that only part of the maturation of DC in response to wt RSV or the ΔNS mutants appeared to be IFN-I-dependent [28]. These results suggest that NS1/2 suppress multiple factors of DC maturation and that this suppression is only partially regulated by secreted IFN-I. For example, we note that the levels of IL-12β and IL-23α mRNA which enhance Th1 and Th17 responses, respectively, were increased 57-759-fold and 49-134-fold, respectively, in DC infected with the ΔNS1/2 virus compared to wt RSV (Fig. 7A, B). The level of expression of each was increased rather than decreased by the IFNAR2 blockade: thus, the expression of IL-12β and IL-23α was suppressed by NS1, but not due to suppressed IFN-I production and signaling.

### The NS1 protein suppresses the levels of IFNγ in co-cultures of RSV-infected DC and T cells

**Figure 8:**
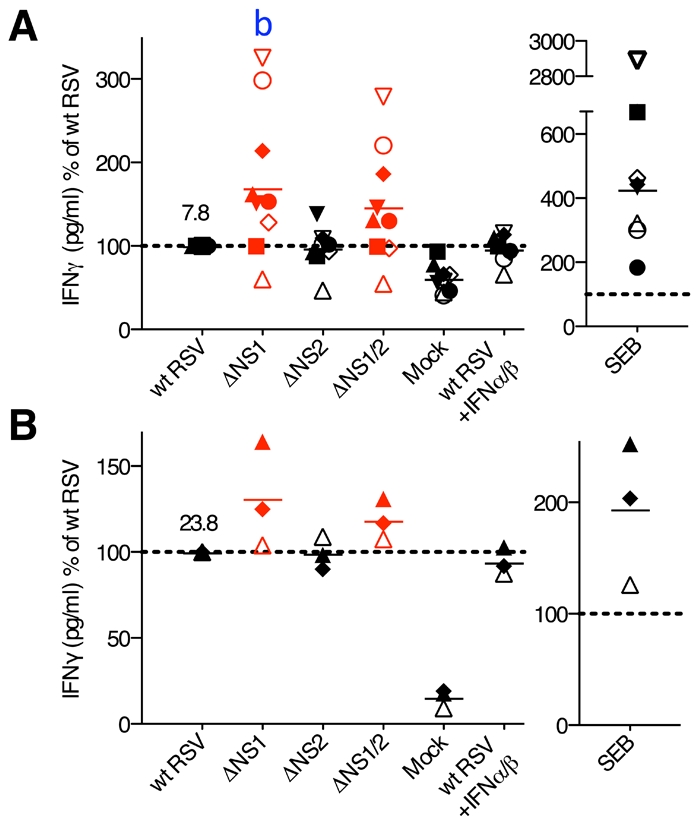
Concentrations of IFNγ in the DC-T cell co-cultures. The CD8+ (A) and CD4+ (B) T cells were co-cultured with DC pre-infected with wt RSV, ΔNS1, ΔNS2 or ΔNS1/2 RSV, and IFNγ concentrations were determined by ELISA. In addition, duplicate of wt RSV co-cultures were prepared containing 133 IU/ml each of exogenously added IFNα2a and IFNβ. The data are normalized to that for wt RSV, which was assigned the value of 100% (dotted line). 9 and 3 donors were analyzed for A and B, respectively, represented by symbols with the means indicated by horizontal bars. In each panel, the mean concentration of IFNγ in pg/ml is indicated for wt RSV. In part A, a significant difference to wt RSV is indicated by the letter b, P<0.01.

Analysis of cytokine expression by flow cytometry reliably determines the percentage of cells that are positive for expression, but since *in vitro* stimulation can induce high levels of expression, the MFI does not necessarily provide an accurate comparison of the level of expression. We therefore also analyzed selected cytokines by ELISA. We performed co-culture of wt and mutant RSV-infected DC with autologous CD8+ or CD4+ T cells, and after 7 days of incubation, analyzed the co-culture media supernatants by ELISA for IFNγ and TNFα (CD8+ T cell co-cultures), or IFNγ, IL-2, IL-4, IL-12 and IL-17 (CD4+ T cell co-cultures). We found that for both CD8+ and CD4+ T cells from most of the donors, the deletion of the NS1 gene was associated with increases in the concentration of secreted IFNγ (mean increases of 70% and 31%, respectively), whereas deletion of both the NS1 and NS2 genes resulted in somewhat lesser increases (Fig. 8). The other cytokines analyzed by ELISA were either not detected or were detected at very low levels (not shown), reflecting a limitation of this method. The addition of exogenous IFN-I at various concentrations (data not shown) or at a single dose (133 IU/ml of IFNα2a and IFNβ each, Fig. 8) during the co-cultures of wt RSV-infected DC with T cells did not significantly affect IFNγ production (Fig. 8 and data not shown). Thus, the presence of the NS1 gene changed the cytokine microenvironment towards reduced secretion of IFNγ, and this effect was not altered by added IFN-I.

## Discussion

**Figure 9:**
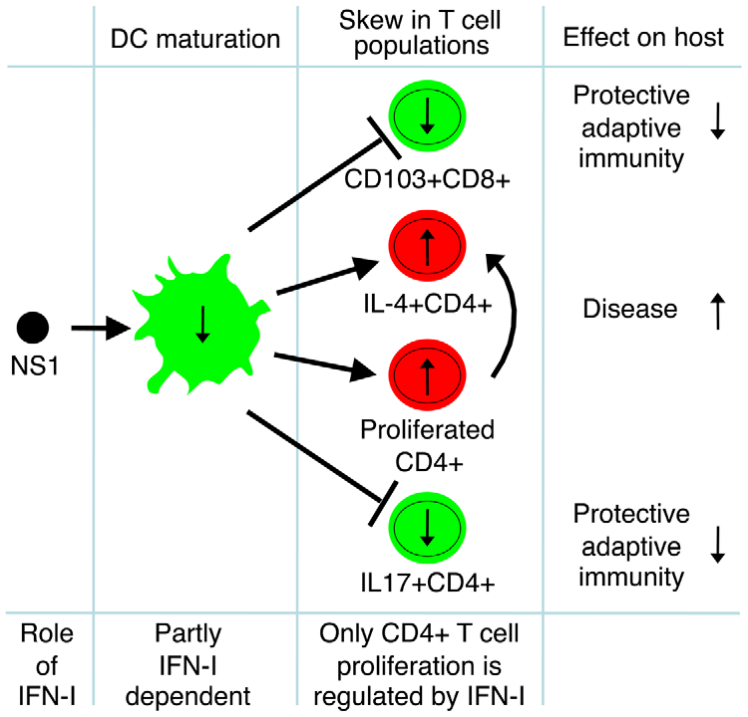
The effects of RSV NS1 protein on T cells. A model of the effects of the RSV NS1 protein on maturation of DC, activation and proliferation of three populations of T cells (CD103+CD8+, IL-4+CD4+ and IL17+CD4+), role of IFN-I, and the effect of NS1-mediated regulation of these cell populations on the host adaptive immunity and RSV disease.

We demonstrated, using primary human immune cells, that the RSV NS1 protein induces quantitative and qualitative deficiencies in adaptive immunity. In particular, the NS1 protein (i) suppresses the CD103+ CD8+ T cell response, (ii) promotes a Th2 response, and (iii) suppresses the Th17 response (Fig. 9). Thus, NS1 suppresses two mechanisms that play a protective role during RSV infection (CD103+ CD8+ T cells and Th17 cells) and stimulates a mechanism contributing to enhanced disease (Th2 cells). A role for CD8+ T cells in restricting and clearing RSV infection is well established [54], [55]. Following infection with RSV or influenza virus, virus-specific CD8+ CTL accumulate in lungs at much greater concentration than in the peripheral blood [30]. Mucosal CD8+CTL, but not their peripheral blood counterparts with the same antigenic specificity, are highly positive for CD103 and have the effector memory phenotype [56]. The presence of CD103 on the surface of lung CD8+ CTL is important for respiratory epithelial cell-specific tropism and cytotoxicity due to expression of E-cadherin by epithelial cells, which specifically binds CD103 [34], [57]. A study with human lung tumor tissues and tumor-specific CD8+ CTL demonstrated that the interaction of CD103 with E-cadherin promotes cytolytic activity by triggering lytic granule polarization and exocytosis [34]. Similarly, it was found that, in human bronchoalveolar lavages, a much greater fraction of both CD4+ and CD8+ T cells expresses CD103, as compared to that in the peripheral blood, and that the CD103 molecule is required for effective lysis of E-cadherin-expressing target cells [35], [57]. Moreover, CD103 plays role in CD8+ T cell retention in human lung tumors by a CCR5-dependent mechanism [37]. Thus, CD103 is a molecule expressed at high levels by mucosal and, in particular, pulmonary CTL, and is associated with their mucosal homing, cytotoxicity against respiratory tract epithelial cells, and their subsequent retention as effector memory T cells. Accumulating evidence thus indicates an important role for CD103 in pulmonary CTL-mediated lysis of virus-infected cells of the respiratory epithelium and thus protection against respiratory viruses. Therefore, the suppression of activation and proliferation of CD103+CD8+ CTL by the RSV NS1 protein demonstrated in this study identifies a new mechanism by which the virus suppresses an important protective component of the adaptive immune response.

Th17 cells were initially found to have a pro-inflammatory effect and to be involved in autoimmune diseases [58]. Subsequent studies elucidated their role in innate and adaptive immune response against pathogens. One of the major functions of IL-17 is the recruitment of neutrophils [59]. Infection of bronchial epithelial cells with rhinovirus, a human respiratory pathogen, demonstrated that the IL-17-mediated recruitment of neutrophils is related to its ability to induce IL-8, which is a neutrophil chemoattractant [60]. Neutrophils play an essential role in antibody-mediated neutralization of influenza virus *in vivo* [61] and play a critical role in the activation of natural killer cells [62]. Using *Listeria monocytogenes,* it was found that Th17 cells are much more strongly induced by the respiratory route of infection than by the intravenous route [63]. It has only recently been found that Th17 cells play a role in protection against viruses. IL-17 produced by Th17 cells facilitates clearance of vaccinia virus infection in mice, most likely by the attraction of neutrophils [64]. Th17 cells have also been shown to protect mice from a lethal dose of influenza virus by a mechanism independent of IFNγ, T cell helper function, or perforin-mediated cytotoxicity, and which possibly involves reduction in the severity of lung damage and/or effect on the rate of lung repair [65]. Th17/Th1 cells exhibit functional properties similar to those of Th17 cells [51]. Thus, the suppression of proliferation of IL-17+ CD4+T cells (Th17 cells) or IL-17+IFNγ+ CD4+ T cells (Th17/Th1 cells) by the RSV NS1 protein found in this study suggests yet another novel mechanism the virus uses to counteract the anti-viral effects of both the adaptive (the Th17 and Th17/Th1 cells) and the innate (secreted IFNγ) components of the immune system.

Severe RSV disease likely is influenced by a variety of factors whose individual importance varies in the heterogeneous human population and likely also with age. One proposed scenario involves a skewing of the Th1/Th2 balance of the virus-specific response towards Th2, which includes down-regulation of the CD8+ CTL response and a B cell switch from synthesis of virus-specific protective IgA and IgG to non-protective IgE (reviewed in [16], [66]). A Th2 bias also has been implicated for a formalin-inactivated RSV vaccine that was evaluated in infants and children in the 1960s and resulted in a greatly increased frequency and severity of disease upon subsequent natural RSV infection [16], [66]. Moreover, a number of studies over the past decade have suggested that severe RSV disease may sometimes be linked to various genetic factors, including those controlling the expression of or response to Th2 cytokines [67]. In particular, polymorphisms leading to an increased activity of IL-4 and IL-13 appeared to be overrepresented in infants and children with severe RSV disease [68], [69], [70], [71]. In the present study, NS1 expressed by wt RSV not only increased the percentage of CD4+ T cells positive for IL-4 (Fig. 5A,B), but also enhanced proliferation of these cells by antagonizing the anti-proliferative effect of IFN-I (Fig. 4). NS1 also depressed the concentration of IFNγ in DC-T cell co-cultures (Fig. 8), thus favoring the Th2 response. These data suggest that the NS1 protein contributes to skewing of the Th1/Th2 balance towards Th2 during the priming of naive T cells or stimulation of memory T cells. The idea that NS1 can skew the T cell response towards Th2 was indicated in previous work. Specifically, when RSV-infected human DC were treated with small interfering RNA targeting the NS1 gene and co-cultivated with heterologous RSV-naïve cord blood CD4+ T cells, the percentage of IFNγ+ cells increased, and that of IL-4+ cells decreased [72]. However, what is surprising in the present study is that these effects do not appear to be dependent on IFN-I production and signaling, as described below.

Since the RSV NS genes are IFN-I antagonists, the most obvious explanation for the effects of NS1 deletion observed in the present study would be that they are mediated by increased IFN-I production and signaling, which are known to affect DC maturation and T cell activation, polarization and proliferation. For example, as noted, NS1 suppresses DC maturation, due in part to inhibition of IFN-I production and signaling [28]. IFN-I has been reported to have stimulatory or inhibitory effects on CD8+ T cell proliferation, depending on the situation (Introduction). Type I and type III IFN have been reported to suppress activation and proliferation of CD4+ T cells during co-culture with RSV-infected DC [15]. IFN-I has been shown to promote a Th1 response by increasing the frequency of human IFNγ-secreting CD4+ Th cells and antagonizing the suppressive effect of IL-4 on IFNγ production [73], [74]. In the present study, one effect indeed did appear to be mediated directly by changes in IFN-I production and IFN-I signaling: specifically, proliferation of total CD4+ T cells was inhibited by IFN-I. However, the other observed effects, namely the suppression of CD103+CD8+ T cells and Th17 cells and enhancement of Th2 cells by NS1, did not appear to be directly mediated by changes in IFN-I production and signaling (Fig. 9). This was suggested by several observations. For example, while both NS1 and NS2, individually and in combination, antagonize IFN-I production and signaling in epithelial cells (Introduction), the effects in the present study were seen only in response to deletion of NS1 and not the NS2 protein. The inclusion of IFNAR2-blocking antibody in the co-cultures provided evidence that the NS1-specific effects on CD103+CD8+ cells, Th17 cells, and Th2 cells were not due to changes in IFN-I production or signaling. This also was indicated in the experiments in which exogenous IFN-I was added to the co-cultures.

An alternative possibility is that these NS1-mediated effects involve suppression of pathways that do not depend on IFN-I production and signaling. As noted, maturation of DCs in response to RSV is partly independent of IFN-I production and signaling [28]. Similarly, Lopez et al. previously demonstrated that the maturation of DC in response to negative strand RNA viruses involves intracellular IFN-I induction pathways, including activation of nuclear factor-kB, but not the released IFN-I or subsequent IFN-I signaling [75]. In the present study, infection of DC with the ΔNS1/2 mutant induced a strong up-regulation in the transcription of multiple genes involved in DC maturation and T cell activation (Fig. 7A, B). Much of this up-regulation was suppressed by wt RSV. The role of IFN-I in this up-regulation was assessed with IFNAR2-blocking antibody. The blockade strongly reduced expression of CXCL9, CXCL10 and RIG-I, which are known to be induced mainly by IFN-I [76], [77], [78]. In contrast, the blockade only partly reduced the up-regulation of several maturation-related markers including CD38, CD40, and CD80 among others. Thus, we suggest that the effects on the T cells observed in the present study result from NS1-mediated suppression of signaling pathways leading to DC maturation, which likely include signaling pathways leading to induction of IFN-I, but are independent of secreted IFN-I and IFN-I signaling. IL-12 and IL-23 also may be involved. IL-12 and IL-23 are involved in polarization of T cell response toward Th1 [79] and maintenance of the Th17 cell population [80], respectively. The expression of each by RSV-infected DC was increased by deletion of NS1/2. This did not appear to be a result of increased IFN-I production and signaling because the IFNAR2 blockade increased rather than decreased expression, indicative of suppression rather than stimulation by IFN-I (Fig. 7A, B). Suppression of IL-12 expression by IFN-I also has previously been reported in humans and mice [81], [82]. NS1 also reduced secretion of IFNγ by both CD8+ and CD4+ T cells. IFNγ promotes the differentiation of Th cells to Th1 and suppresses their development to Th2 phenotype, and IFNγ secreted by CD8+ T cells prevents Th2-driven pathology during RSV infections [83], [84]. Thus, suppression of IFNγ secretion by CD4+ T cells, which was not reversed by exogenous IFN-I, may be involved in the Th2-promoting effect of the NS1 protein demonstrated in this study.

In conclusion, the data presented in this study suggest that the RSV NS1 protein has quantitative and qualitative effects on the adaptive immune response, thus promoting infection and disease. The experimental system used in this study involved human primary cells from adult donors and thus represents a model of the immune system of RSV-immune children and adults for investigation of the effects of immunomodulating RSV proteins on the virus-specific memory T cells. However, the pattern of immune response to RSV depends on age and is biased towards Th2 in neonates [85]. Therefore, it is possible that, in RSV-naïve individuals, the three major effects of the NS1 protein, especially that of enhanced Th2 activity, might be even more pronounced than in adults.

## Materials and Methods

### Virus propagation and purification

Virus growth and purification were performed as described previously [28], [86]. Briefly, Vero cells were maintained in Opti-MEM I mefdium (Invitrogen, Carlsbad, CA) supplemented with 5% heat-inactivated fetal bovine serum (FBS) (HyClone, Logan, UT), 2 mM L-glutamine, 100 IU/ml penicillin and 100 µg/ml streptomycin sulfate (Invitrogen). Recombinant wild type (wt) RSV strain A2 and RSV A2 deletion mutants lacking NS1 (ΔNS1), NS2 (ΔNS2) or both NS1 and NS2 (ΔNS1/2) genes, all expressing enhanced green fluorescent protein to monitor DC infection, described previously [28], were propagated in Vero cells. Virus-infected Vero cell supernatants were harvested and viruses were purified by sucrose gradient centrifugation. In order to eliminate sucrose from purified viruses, virus bands were diluted with Advanced RPMI 1640 medium with 2 mM L-glutamine (Invitrogen), and the viruses were pelleted at 8,000 x g for 2 h at 4°C, followed by re-suspension in the same medium. Aliquots of the viruses were stored at −80°C and titers were determined by plaque assay as previously described [87].

### Preparation of DC and T lymphocytes

Elutriated monocytes obtained from healthy adult donors according to an approved clinical protocol were provided by the NIH blood bank and were differentiated into monocyte-derived DC cultures as described previously [28]. Briefly, CD14+ monocytes were purified by positive selection using anti-CD14 monoclonal antibody-conjugated magnetic microbeads and an Automacs separator (Miltenyi Biotech, Auburn, CA), and were cultured with granulocyte-macrophage colony-stimulating factor (GM-CSF) and IL-4. Cultures were incubated at 37°C for 7 days during which time the cells developed phenotypic features of immature DC (CD1a^+^ CD14^low^ CD38^low^ CD11c^high^) [88].

Autologous elutriated lymphocytes from the same donors were also provided by the NIH blood bank and were purified by density centrifugation in Ficoll-Hypaque (Cellgro-Mediatech, Manassas, VA), harvested and washed in Advanced RPMI 1640 medium (Invitrogen) supplemented with 10% FBS (HyClone) and 2 mM L-glutamine (Invitrogen). Lymphocytes were cryopreserved in Advanced RPMI 1640 medium containing 10% FBS (HyClone), 2 mM L-glutamine (Invitrogen), 10% dimethyl sulfoxide (DMSO) (Cellgro-Mediatech) at a cell density of 4×10^7^ cell/ml. Before use, the lymphocytes were thawed and cultured overnight in Advanced RPMI 1640 medium supplemented with 10% human serum (Gemini Bio-products, West Sacramento, CA), 2 mM L-glutamine, 200 IU/ml penicillin, and 200 µg/ml streptomycin sulfate (Invitrogen). The next day, CD8+ T cells were purified from lymphocytes by negative selection with an Automacs separator using a primary cocktail of antibodies conjugated to biotin and secondary anti-biotin antibody conjugated to magnetic microbeads to deplete non-CD8+ T cells, including CD4+ T cells, γ/δ T cells, B cells, NK cells, DC, monocytes, granulocytes, and erythroid cells. The cells were analyzed for purity by staining with anti-CD8 phycoerythrin (PE)-conjugated antibodies (BD Biosciences, San Jose, CA) and flow cytometry. CD4+ T cells were isolated from lymphocytes by positive selection with an Automacs using anti-CD4 monoclonal antibody-coated magnetic microbeads (Fig. 1A). The purity of the cells was assessed by staining with anti-CD4 fluorescein isothiocyanate (FITC)-conjugated antibodies (BD Biosciences) and flow cytometry. The purity of CD8+ and CD4+ T cells was 85–95% and >98%, respectively. Purified CD8+ and CD4+ T cells were labeled with CFSE (Invitrogen) as per the manufacturer's instructions, in order to monitor cell proliferation as assessed by reduction in the CFSE fluorescence intensity due to cell divisions.

### Infection of DC and co-culture with T cells

On day 7 of culture, when monocytes had differentiated into DC, they were harvested and labeled either with CellVue Lavender (Molecular Technologies, West Chester, PA) or 7-hydroxy-9H-(1,3-dichloro-9,9-dimethylacridin-2-one) (DDAO) (Invitrogen) fluorescent tracers, as per manufacturer's instructions, for co-culture with CD8+ and CD4+ T cells, respectively (Fig. 1A). Following labeling, DC were inoculated with wt RSV, ΔNS1, ΔNS2 or ΔNS1/2 RSV at MOI of 2 PFU/cell, mock treated or treated with Staphylococcal Enterotoxin B (SEB) (Sigma-Aldrich, St. Louis, MO) at 1 µg/ml. After 4 h of incubation, DC were washed to remove virus inoculum and were co-cultured with purified autologous CFSE-labeled CD8+ or CD4+ T cells at a DC: lymphocyte ratio of 1∶10 (2×10^5^ DC: 2×10^6^ lymphocytes) in 2 ml of Advanced RPMI 1640 medium (Invitrogen) containing 10% human serum (Gemini Bio-Products), 2 mM L-glutamine, 200 IU/ml penicillin, and 200 µg/ml streptomycin sulfate (all Invitrogen) in 12-well plates. Co-cultures were incubated at 37°C for 7 days. This is based on our previous finding that proliferation was minimal by day 4 and requires 7 days to be substantial [89]. On day 5, 1 ml of fresh medium was added to the 2 ml of pre-existing medium of each co-culture. Since the deletion of NS1 protein significantly increases the production of IFN-I from infected DC, which may in turn be responsible for the T cell phenotypes observed in our experiments, a control was included where exogenous IFNα2a and IFNβ (PBL Interferon Source, Piscataway, NJ) were added at 133 IU/ml each to co-culture of DC pre-infected with wt RSV with CD4+ as well as CD8+ T cells to monitor such effects. The quantity of IFN-I added to this control was based on our previous studies [28] and represents the average amount produced by human monocyte-derived DC on infection with the viruses lacking NS1.

For IFN-I receptor blocking experiments, mouse monoclonal anti-human IFNAR2-neutralizing antibody (PBL Interferon Source) or its isotype control antibody (R&D Systems, Minneapolis, MN) was added to the purified CD8+ or CD4+ T cells at a final concentration of 60 µg/ml and incubated for 1 h at 37°C. We chose to use an IFNAR2-blocking antibody because the antiviral activity of IFN-I correlates well with signaling through IFNAR2 rather than IFNAR1 [90]. This mixture was then combined with autologous DC that had been pre-infected with wt RSV or ΔNS1/2 RSV at MOI of 2 PFU/cell or mock infected as described, resulting in final antibody concentration of 30 µg/ml. The co-cultures were incubated at 37°C for 7 days. In another format, the antibody was added to DC for 2 h, followed by infection and additional incubation for 48 h, wash and co-cultivation with T cells as above. To determine the effect of IFN-I, an equal mixture of IFNα2a and IFNβ (PBL Interferon Source) was added exogenously to a final concentration of 25, 50, 100, 133, 200, 400, 800, or 1,600 IU/ml each to the co-cultures of DC pre-infected with wt RSV and CD8+ or CD4+ T cells. The co-cultures were incubated at 37°C for 7 days.

### Staining of CD8+ and CD4+ T cells

After 7-days of co-culture, medium supernatants were collected for cytokine assay. The co-cultures were then prepared for intracellular cytokine staining by stimulation with phorbol-12-myristate-13-acetate (PMA) (Sigma Aldrich, St. Louis, MO) at 20 ng/ml and ionomycin (EMD Chemicals, Gibbstown, NJ) at 1 µM in the presence of brefeldin A (Sigma Aldrich) at 10 µg/ml, and were incubated at 37°C for 6 h; 5 min before the harvest, DNase I (Calbiochem, Gibbstown, NJ) was added at 150 µg/ml. Following harvest, cells were stained with Live/Dead Fixable Blue Dead Cell Stain (Invitrogen) to discriminate dead cells by flow cytometry. CD8+ T cells were also stained with PE-labeled anti-CD103 antibodies (BD Biosciences), peridinin chlorophyll protein (PerCP-Cy5.5)-labeled anti-CD107a antibodies (Biolegend, San Diego, CA), and pacific orange-labeled anti-CD8 antibodies (Invitrogen). After surface staining, cells were fixed and permeabilized using fixation/permeabilization kit (BD Biosciences) as per the manufacturer's instructions. Cells were then blocked and permeabilized by overnight incubation in phosphate buffered saline containing 0.1% saponin and 5% non-fat dry milk. CD8+ T cells were then stained for intracellular cytokines and cell markers with allophycocyanin-Alexa Fluor 750 (APC-Cy7)-labeled anti-IFNγ (Invitrogen), PE-Cy7-labeled anti- TNFα (BD Biosciences), APC-labeled anti-IL-2 (BD Biosciences), and PE Texas Red-labeled anti-CD3 antibodies (Beckman Coulter, Brea, CA). CD4+ T cells were stained for intracellular cytokines with APC-Cy7-labeled anti-IFNγ (Invitrogen), PE-labeled anti-IL-4 (BD Biosciences), PerCP-Cy5.5-labeled anti-IL-17A (eBioscience, San Diego, CA), PE-Cy7-labeled anti- TNFα (BD Biosciences), and Alexa-Fluor 680-labeled anti-IL-2 antibodies (Biolegend). The stained cells were suspended in staining buffer [phosphate buffered saline, 1% FBS (HyClone)] for flow cytometry analysis.

### Multi-color flow cytometry

Ten-parameter and 9-parameter flow cytometric analysis was performed for CD8+ and CD4+ T cells, respectively, on a Becton Dickinson LSR II flow cytometer (BD Biosciences). Data compensation was performed using mouse and rat CompBeads compensation beads (BD Biosciences) stained with individual antibodies used in the multicolor analysis. Data acquired from the T cells stained only with CFSE and that from DC stained only with CellVue Lavender dye or DDAO were used for compensations involving these fluorochromes. The data were analyzed with FlowJo software version 8.8.6 (Tree Star, Ashland, OR). The data acquired from stained compensation beads and cells were used for automatic compensation using the FlowJo Compensation Wizard. In order to determine the accuracy of compensation between overlapping spectra and to define the positive cells, fluorescence-minus-one (FMO) staining controls were included. The FMO controls included staining of CD4+ T cells, derived from co-cultures with wt RSV-infected DC, with fluorochrome-conjugated antibodies for all cytokines omitting one antibody conjugate per control. The gating strategy followed to obtain proliferated CD8+ or CD4+ T cells for analyses is shown in Fig. 1B. Briefly, the compensated data were gated at UV Live/Dead stain versus CellVue Lavender dye or DDAO to eliminate dead T cells and all DC, and to obtain live T cells. Live T cells were then gated at UV Live/Dead dye versus CD3 to obtain live CD3+ T cells, which were then gated at forward scatter area versus forward scatter height to eliminate cell doublets and clusters and to identify single cells for analyses. Single cells thus identified were again gated at forward scatter area versus side scatter area to exclude any debris and determine cell size and granularity. These cells were then finally gated at CD3 versus CFSE to identify proliferated CD3+ T cells as assessed by reduction in fluorescence intensity of CFSE as a result of cell divisions. For CD8+ T cells, an additional gate was drawn after gate 2 at CD3 versus CD8 to identify CD3+ CD8+ T cells. The proliferated cells thus obtained were further gated for individual cytokines and activation markers including CD103, CD107a, IFNγ, TNFα and IL-2 for CD8+ T cells, and IFNγ, IL-4, IL-17, TNFα and IL-2 for CD4+ T cells. The FMO controls demonstrated that cells stained with all but one cytokine antibody were indeed negative for that cytokine (Fig. S2). This suggests that the electronic compensations applied to the data were accurate, and that the cells found positive for a particular cytokine were indeed a result of specific staining with the corresponding antibody conjugate and were not false positives due to fluorescence contribution from the overlapping spectra. Proliferated T cells gated positive for individual cytokines were analyzed by Boolean gating to obtain frequencies for 32 possible cytokine combinations both positive and negative for individual cytokines using FlowJo software. The identified cell populations were further analyzed using Pestle and Spice software (version 4.2.3; Mario Roederer and Joshua Nozzi, Vaccine Research Center and Bioinformatics and Scientific IT Program, NIAID, NIH, Bethesda, MD). Data obtained by Boolean gating were normalized to wt RSV and plotted for graphical representation using Prism 5 software (GraphPad software Inc., La Jolla, CA).

### Analysis of mRNA isolated from RSV-infected DC and T cells by QRT-PCR

DC were treated with the IFNAR2 blocking antibody or its isotype control for 2 h and then infected with wt RSV or ΔNS1/2 RSV, followed by 48 h-long incubation and analysis of maturation markers by QRT-PCR. Total RNA was extracted from DC using RNeasy kit (Qiagen, Valencia, CA) and used to synthesize cDNA using Superscript III reverse transcriptase kit (Invitrogen) as per manufacturer's protocol. The cDNA thus obtained was used to analyze 49 genes (the full gene names and the NCBI gene reference numbers are provided in Table S1) in triplicate by QRT-PCR on a TaqMan micro fluidic gene card (Applied Biosystems, Foster City, CA) using TaqMan universal PCR master mix (Applied Biosystems). The 18S ribosomal RNA was included on the gene card in triplicate and used as an endogenous control for analysis. PCR was run on 7900HT fast real-time PCR system (Applied Biosystems). The threshold cycle (Ct) values obtained for DC infected with wt RSV without any antibody treatment were used as the calibrator for calculating the fold change in gene expression for the rest of the treatments. The entire experiment was performed four times using DC prepared from four individual donors. The expression fold change values were converted to log_2_ and analyzed with Genesis software version 5 (Alexander Sturn, Institute for Genomics and Bioinformatics, Graz University of Technology, Petersgasse, Austria) to perform the hierarchical clustering of data and generate a heat map. The fold changes for CXCL9, CXCL10, RIG-I, CD38, CD40, CD80, IL-12β, and IL-23α were also plotted for graphical representation using Prism 5 software (GraphPad software Inc.).

2×10^6^ CD8+ or CD4+ T cells purified from elutriated lymphocytes as above were treated with IFNAR2 antibody at concentrations 0.03, 0.3, 3, or 30 µg/ml, plated in 12-well plates and incubated for 2 h at 37°C. Next, IFNα2a was added to all cells at 100 IU/ml, followed by incubation at 37°C. Cells were harvested at 3, 6, and 18 h later, and total RNA was isolated using RNeasy kit (Qiagen, CA). The mRNA expression of MX1 and ISG56 was investigated by QRT-PCR using TaqMan gene expression assay primers and probe set and one step RT-PCR master mix (Applied Biosystems). The 18S ribosomal RNA was quantitated for all samples in triplicate, using TaqMan primers and probe, and was used as endogenous control. The reactions were run in triplicate in ABI 7900HT fast real-time PCR system (Applied Biosystems) and analyzed with SDS 2.3 and RQ Manager 1.2 softwares (Applied Biosystems). Quantitation of MX1 expression in cells treated with IFNAR2 antibody and IFNα2a was analyzed relative to the calibrator, i.e., cells treated with IFNα2a in the absence of IFNAR2 antibody. The data is presented as MX1 expression relative to the calibrator.

### Enzyme linked immunosorbent assays (ELISA)

Co-culture medium supernatants were analyzed for various cytokines by ELISA. Supernatants from CD8+ T cells were analyzed for IFNγ and TNFα while those from CD4+ T cells were assayed for IFNγ, IL-4, IL-17, IL-2 and IL-12. All ELISAs were performed with Quantikine colorimetric sandwich ELISA kits (R&D Systems, Minneapolis, MN). DC supernatants were analyzed for IFNα using IFNα multi-subtype ELISA kit (PBL Interferon Source).

### Statistical analyses

All data involving the comparison of wt RSV with its NS1 and/or NS2 deletion mutants were analyzed for statistical significance using repeated measures ANOVA with Tukey's multiple comparison post test (GraphPad Prism 5 software). In the experiments involving blocking of IFNAR2 or adding exogenous IFN-I, paired one-tailed T-test was used (GraphPad Prism 5 software). For all statistical analyses data were considered significant at P<0.05.

## Supporting Information

Figure S1 The NS1 protein reduces the MFI of CD103 in CD8+ T cells (A), and increases the MFI of IL-4 in CD4+ T cells (B). For A and B, the MFI is shown for proliferated cells positive for the indicated markers of activation (shown on the top of each panel) after their co-cultivation with autologous DC pre-infected with the ΔNS1, ΔNS2 or ΔNS1/2 RSV deletion mutants, as compared to cells from the same donors co-cultivated with DC pre-infected with wt RSV, for which a value of 100% (dotted line) has been assigned. Each symbol represents an individual donor and the horizontal bars indicate mean values. For the ΔNS1 and ΔNS1/2 viruses, significant differences to wt RSV are indicated by letters: a, P<0.05; b, P<0.01.
(0.25 MB TIF)

Figure S2 Fluorescence-minus-one (FMO) controls for multi-color staining of CD4+ T cells. A. Flow cytometry of cells stained for CFSE and all cytokines except the one indicated at the top of each panel. The figure demonstrates that omitting the antibody for each cytokine results in a lack of detection of that cytokine in the proliferating cells. B. Flow cytometry of cells stained for all cytokines except IFNγ or IL-4. Exclusion of staining of IFNγ or IL-4 results in lack of their detection, and the population positive for both IFNγ and IL-4 (the left panel) is detected only when cells are stained with both the corresponding antibodies. The data suggest that IFNγ+IL-4+ cells indeed exist as a double positive phenotype and are not a result of spectral overlap. C. Flow cytometry analysis of IFNγ or IL-17 FMO as performed in part B.
(0.66 MB TIF)

Table S1 Genes analyzed for transcription by QRT-PCR using microfluidic gene card. The gene expression results are depicted in figure 7.
(0.09 MB DOC)
