# Supplementary figures and images for: Respiratory Syncytial Virus Interferon Antagonist NS1 Protein Suppresses and Skews the Human T Lymphocyte Response
Source: PLoS Pathog. 2011 Apr 21;7(4):e1001336. doi: 10.1371/journal.ppat.1001336 (PMC3080852; doi:10.1371/journal.ppat.1001336)

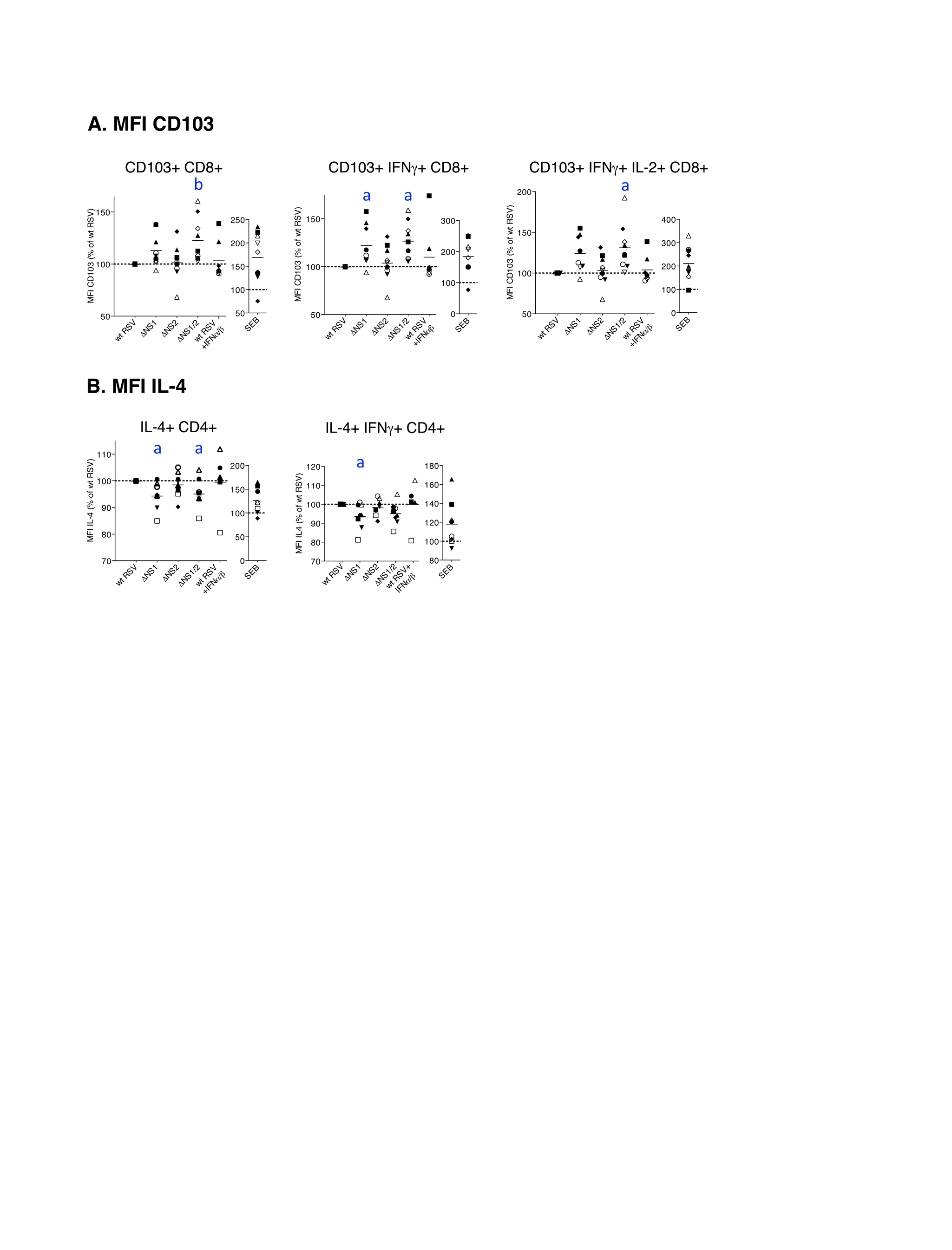

Supplement: Figure S1 — The NS1 protein reduces the MFI of CD103 in CD8+ T cells (A), and increases the MFI of IL-4 in CD4+ T cells (B). For A and B, the MFI is shown for proliferated cells positive for the indicated markers of activation (shown on the top of each panel) after their co-cultivation with autologous DC pre-infected with the ΔNS1, ΔNS2 or ΔNS1/2 RSV deletion mutants, as compared to cells from the same donors co-cultivated with DC pre-infected with wt RSV, for which a value of 100% (dotted line) has been assigned. Each symbol represents an individual donor and the horizontal bars indicate mean values. For the ΔNS1 and ΔNS1/2 viruses, significant differences to wt RSV are indicated by letters: a, P<0.05; b, P<0.01. (0.25 MB TIF) [file ppat.1001336.s001.tif]

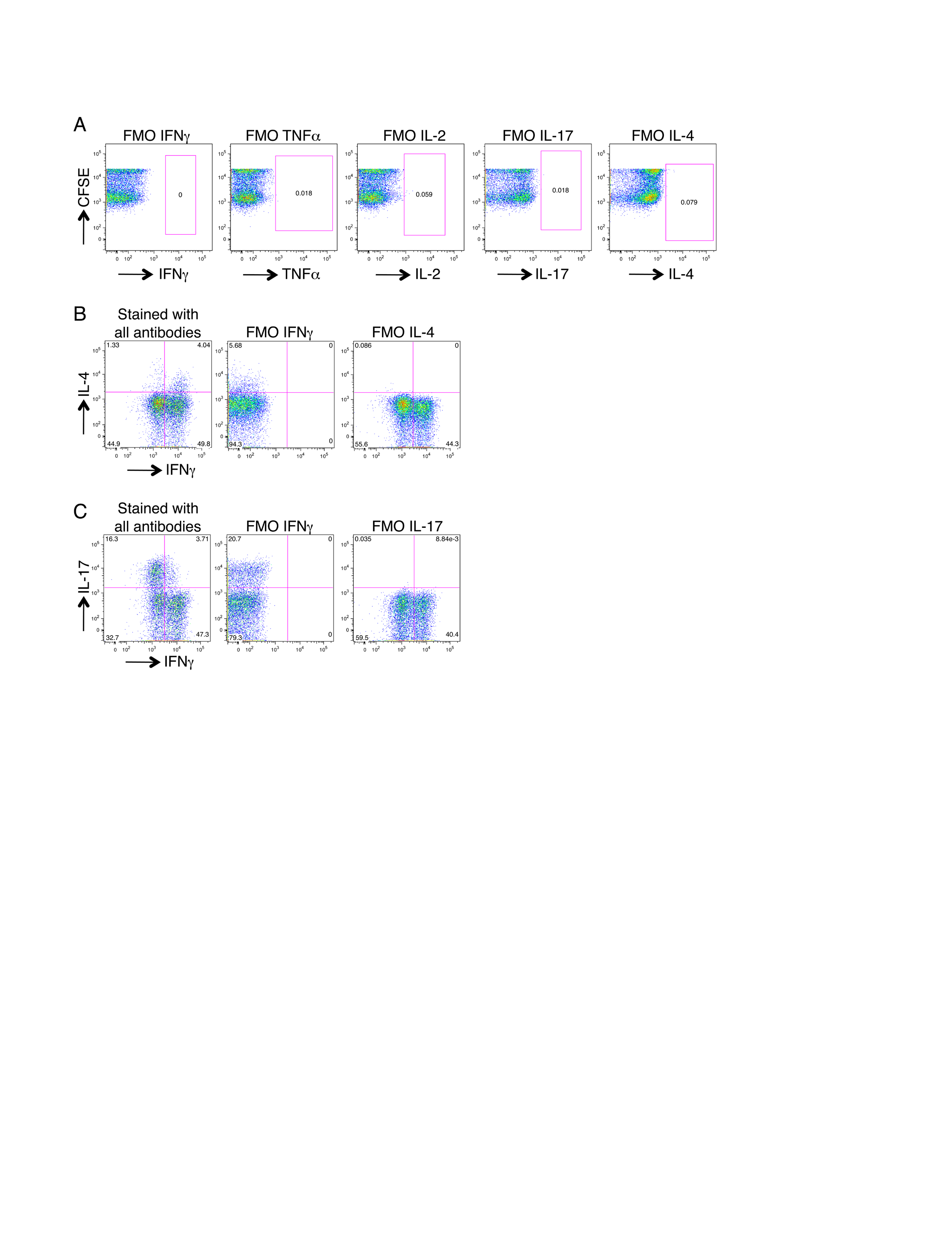

Supplement: Figure S2 — Fluorescence-minus-one (FMO) controls for multi-color staining of CD4+ T cells. A. Flow cytometry of cells stained for CFSE and all cytokines except the one indicated at the top of each panel. The figure demonstrates that omitting the antibody for each cytokine results in a lack of detection of that cytokine in the proliferating cells. B. Flow cytometry of cells stained for all cytokines except IFNγ or IL-4. Exclusion of staining of IFNγ or IL-4 results in lack of their detection, and the population positive for both IFNγ and IL-4 (the left panel) is detected only when cells are stained with both the corresponding antibodies. The data suggest that IFNγ+IL-4+ cells indeed exist as a double positive phenotype and are not a result of spectral overlap. C. Flow cytometry analysis of IFNγ or IL-17 FMO as performed in part B. (0.66 MB TIF) [file ppat.1001336.s002.tif]
