# Supplementary material for: Respiratory Syncytial Virus Interferon Antagonist NS1 Protein Suppresses and Skews the Human T Lymphocyte Response
Source: PLoS Pathog. 2011 Apr 21;7(4):e1001336. doi: 10.1371/journal.ppat.1001336 (PMC3080852; doi:10.1371/journal.ppat.1001336)
Supplement: Table S1 — Genes analyzed for transcription by QRT-PCR using microfluidic gene card. The gene expression results are depicted in figure 7. (0.09 MB DOC) [file ppat.1001336.s003.doc]

**Supporting Table 1.**

| **Gene Symbol** | **Gene Name** | **NCBI Gene Reference** |
| --- | --- | --- |
| MYD88 | Myeloid differentiation primary response gene (88) | NM_002468.4 |
| JAK1 | Janus kinase 1 | NM_002227.2 |
| 18S | Eukaryotic 18S rRNA | X03205.1 |
| CD80 | CD80 molecule | NM_005191.3 |
| CD209 | CD209 molecule | NM_001144893.1 |
| IFNα1 | Interferon, alpha 1 | NM_024013.1 |
| IL18 | Interleukin 18 (interferon-gamma-inducing factor) | NM_001562.2 |
| DDX58 (RIG-I) | DEAD (Asp-Glu-Ala-Asp) box polypeptide 58 | NM_014314.3 |
| CD14 | CD14 molecule | NM_001040021.1 |
| STAT1 | Signal transducer and activator of transcription 1, 91kDa | NM_139266.2 |
| CD86 | CD86 molecule | NM_175862.3 |
| CD274 | CD274 molecule | NM_014143.2 |
| IFNα2 | Interferon, alpha 2 | NM_000605.3 |
| IFIH1 | Interferon induced with helicase C domain 1 | NM_022168.2 |
| TICAM1 | Toll-like receptor adaptor molecule 1 | NM_182919.2 |
| STAT3 | Signal transducer and activator of transcription 3 (acute-phase response factor) | NM_213662.1 |
| HLA-A | Major histocompatibility complex, class I, A | NM_002116.6 |
| PDCD1LG2 | Programmed cell death 1 ligand 2 | NM_025239.3 |
| IFNβ1 | Interferon, beta 1, fibroblast | NM_002176.2 |
| IL1β | Interleukin 1, beta | NM_000576.2 |
| IL23α | Interleukin 23, alpha subunit p19 | NM_016584.2 |
| IRF1 | Interferon regulatory factor 1 | NM_002198.2 |
| NFκB1 | Nuclear factor of kappa light polypeptide gene enhancer in B-cells 1 | NM_001165412.1 |
| HLA-B | Major histocompatibility complex, class I, B | D83043.1 |
| IL27 | Interleukin 27 | NM_145659.3 |
| TLR3 | Toll-like receptor 3 | NM_003265.2 |
| IRF3 | Interferon regulatory factor 3 | NM_001571.4 |
| NFκB2 | Nuclear factor of kappa light polypeptide gene enhancer in B-cells 2 (p49/p100) | NM_001077493.1 |
| HLA-C | Major histocompatibility complex, class I, C | NM_002117.4 |
| IL6 | Interleukin 6 (interferon, beta 2) | NM_000600.2 |
| IL28A | Interleukin 28A (interferon, lambda 2) | NM_172138.1 |
| TLR4 | Toll-like receptor 4 | NM_138554.3 |
| IRF7 | Interferon regulatory factor 7 | NM_001572.3 |
| CD38 | CD38 molecule | NM_001775.2 |
| HLA-DPA1 | Major histocompatibility complex, class II, DP alpha 1 | NM_033554.2 |
| IL10 | Interleukin 10 | NM_000572.2 |
| IL29 | Interleukin 29 (interferon, lambda 1) | NM_172140.1 |
| TLR7 | Toll-like receptor 7 | NM_016562.3 |
| SOCS1 | Suppressor of cytokine signaling 1 | NM_003745.1 |
| CD40 | CD40 molecule, TNF receptor superfamily member 5 | NM_152854.2 |
| HLA-DQB1 | Major histocompatibility complex, class II, DQ beta 1 | NM_002123.3 |
| IL12α | Interleukin 12A (natural killer cell stimulatory factor 1, cytotoxic lymphocyte maturation factor 1, p35) | NM_000882.2 |
| TNF | Tumor necrosis factor (TNF superfamily, member 2) | NM_000594.2 |
| TLR8 | Toll-like receptor 8 | AF246971.1 |
| SARM1 | Sterile alpha and TIR motif containing 1 | NM_015077.2 |
| ICAM1 | Intercellular adhesion molecule 1 | NM_000201.2 |
| IL12β | Interleukin 12B (natural killer cell stimulatory factor 2, cytotoxic lymphocyte maturation factor 2, p40) | NM_002187.2 |
| TGFβ1 | Transforming growth factor, beta 1 | NM_000660.4 |
| CXCL9 | chemokine (C-X-C motif) ligand 9 | NM_002416.1 |
| CXCL10 | chemokine (C-X-C motif) ligand 10 | NM_001565.2 |
